# Updated Global Burden of Cholera in Endemic Countries

**DOI:** 10.1371/journal.pntd.0003832

**Published:** 2015-06-04

**Authors:** Mohammad Ali, Allyson R. Nelson, Anna Lena Lopez, David A. Sack

**Affiliations:** 1 Department of International Health, Johns Hopkins Bloomberg School of Public Health, Baltimore, Maryland, United States of America; 2 University of the Philippines Manila-National Institutes of Health, Manila, Philippines; Emory University, UNITED STATES

## Abstract

**Background:**

The global burden of cholera is largely unknown because the majority of cases are not reported. The low reporting can be attributed to limited capacity of epidemiological surveillance and laboratories, as well as social, political, and economic disincentives for reporting. We previously estimated 2.8 million cases and 91,000 deaths annually due to cholera in 51 endemic countries. A major limitation in our previous estimate was that the endemic and non-endemic countries were defined based on the countries’ reported cholera cases. We overcame the limitation with the use of a spatial modelling technique in defining endemic countries, and accordingly updated the estimates of the global burden of cholera.

**Methods/Principal Findings:**

Countries were classified as cholera endemic, cholera non-endemic, or cholera-free based on whether a spatial regression model predicted an incidence rate over a certain threshold in at least three of five years (2008-2012). The at-risk populations were calculated for each country based on the percent of the country without sustainable access to improved sanitation facilities. Incidence rates from population-based published studies were used to calculate the estimated annual number of cases in endemic countries. The number of annual cholera deaths was calculated using inverse variance-weighted average case-fatality rate (CFRs) from literature-based CFR estimates. We found that approximately 1.3 billion people are at risk for cholera in endemic countries. An estimated 2.86 million cholera cases (uncertainty range: 1.3m-4.0m) occur annually in endemic countries. Among these cases, there are an estimated 95,000 deaths (uncertainty range: 21,000-143,000).

**Conclusion/Significance:**

The global burden of cholera remains high. Sub-Saharan Africa accounts for the majority of this burden. Our findings can inform programmatic decision-making for cholera control.

## Author Summary

The global burden of cholera is largely unknown because the majority of cases are not reported. The low reporting can be attributed to limited capacity of epidemiological surveillance and laboratories, as well as social, political, and economic disincentives for reporting. We previously estimated 2.8 million cases and 91,000 deaths annually due to cholera in 51 endemic countries. A major limitation in our previous estimate was that the endemic and non-endemic countries were defined based on the countries’ reported cholera cases. If a country did not report cases even though the country had cholera, the country was classified as cholera free. This time we addressed this limitation by using a spatial modelling technique, which helped us define the cholera-endemic countries based on access to improved water and sanitation in the country as well as cholera incidence in neighboring countries. Our new estimate illustrates 2.9 million of cases and 95,000 deaths in 69 endemic countries, with the majority of the burden in Sub-Saharan Africa. The sustained high burden of cholera points to the necessity for integrated and improved control efforts, and these findings may help programmatic decision-making for controlling the disease in endemic countries.

## Introduction

Since the early 1800s, pandemics of cholera have affected millions, with the seventh still ongoing since 1961 [1]. Access to safe water and improved sanitation facilities has eliminated cholera transmission of *Vibrio cholerae*, the causative agent, in high-income countries. However, the bacteria continue to afflict millions of people in less developed countries where improved water and sanitation infrastructure are not widely available.

The actual global burden of cholera is largely unknown as the vast majority of cases are not reported. The World Health Organization (WHO) maintains a repository of reported cases and deaths, and publishes annual statistics in the *Weekly Epidemiological Record* (WER). However, the WHO estimates that only 5–10% of the cases occurring annually are officially reported [2]. This low reporting efficiency is due to a combination of factors including limited capacity of epidemiological surveillance systems and laboratories, and social, political and economic disincentives for reporting [3–5].

Previously, the global burden of cholera was estimated at 2.8 million cases of cholera annually, with 91,000 deaths [6]. A major limitation of the previous estimate was that the endemic and non-endemic countries were defined solely based on the countries’ reported cholera cases. If a country did not report cases but did, in fact, have cases of cholera, the country was classified as cholera-free. Furthermore, the data used for the previous estimate were from 2000–2008, prior to the re-appearance of cholera in the Americas.

An update of the global burden of cholera is needed to assist public health practitioners and policy-makers in cholera control efforts. In 2010, a large outbreak of cholera affecting the Americas changed the epidemiology of the disease [7], and the World Health Organization recommended the inclusion of oral cholera vaccines (OCVs) as part of an integrated strategy to control cholera [8, 9]. In 2013, a stockpile was created to enable the use of OCV in outbreaks and support for the stockpile was provided by Gavi [10].

This study provides an updated global burden of cholera and addresses some of the limitations of the previous global burden study. It differs from the previous study in that we predicted the presence of cholera cases at the country-level on an annual basis using a multivariable spatial regression model to define the status of a country: endemic, non-endemic, and cholera free. The model allowed for prediction of cholera incidence rate based on the water and sanitation conditions of each country as well as the cholera incidence rate in the 1^st^ order neighboring countries.

## Methods

### Data Sources

A systematic search of all publicly available cholera case and fatality data was conducted. The sources of data included reports to the WHO published annually in the Weekly Epidemiology Records, PubMed, the Global Infectious Disease and Epidemiology Network (GIDEON), the Program for Monitoring Emerging Diseases (ProMED), and Google. The primary sources of data were the WHO’s 2008–2012 WER Annual Cholera Global Surveillance Summaries, which aggregate all cholera cases and fatalities reported to the WHO on an annual basis. This was supplemented with data from GIDEON. The data collected by GIDEON use computer macros which regularly scan source lists including Medline, ProMED, WHO, CDC, national Ministry of Health standard publications, and relevant peer-reviewed journals. Systematic searches of ProMED, PubMed, and Google were also performed using the search terms “cholera” and “acute watery diarrhea.” The use of multiple sources ensured that all available data were captured from WHO reports, Ministries of Health, research bodies or the media. For annual cases, the source with the highest number of reported cases was used as the final case count. Imported cases were excluded from the case count. The source with the highest number of reported deaths was used as the final death count. All counts were aggregated at the country-level with the exception of India, China, and Indonesia, for which counts were aggregated at the sub-national level. These three countries were analyzed at the sub-national level due to their large population and geographic size, the spatially heterogeneous nature of cholera epidemiology, and the availability of cholera reports at the sub-national level. The 14 states in India, 5 provinces in China, and 4 provinces in Indonesia that reported cholera cases during the study period were included in the analysis.

The population data of a country was collected from the United Nations Development Program (UNDP) *World Population Prospects*: *The 2012 Revision* [11]. This source was selected as it had the most current population estimates (2010). The data on accessibility to improved water and sanitation facility were collected from UNICEF’s *State of the World’s Children Report 2013*, which had the most current estimates (2010) [12]. Use of improved sanitation facilities (%) was defined as the “percentage of the population using any of the following sanitation facilities, not shared with other households: flush or pour-flush latrine connected to a piped sewerage system, septic tank or pit latrine; ventilated improved pit latrine; pit latrine with a slab; covered pit; composting toilet. Access to improved drinking water source was defined as the percentage of the population using any of the following as the main drinking water source: drinking water supply piped into dwelling, plot, yard or neighbor’s yard; public tap or standpipe; tube well or borehole; protected dug well; protected spring; rainwater; bottled water plus one of the previous sources as a secondary source [12]. For countries with missing data, WHO estimates from 2006 [13] were used.

### Country Stratification

**Fig 1:**
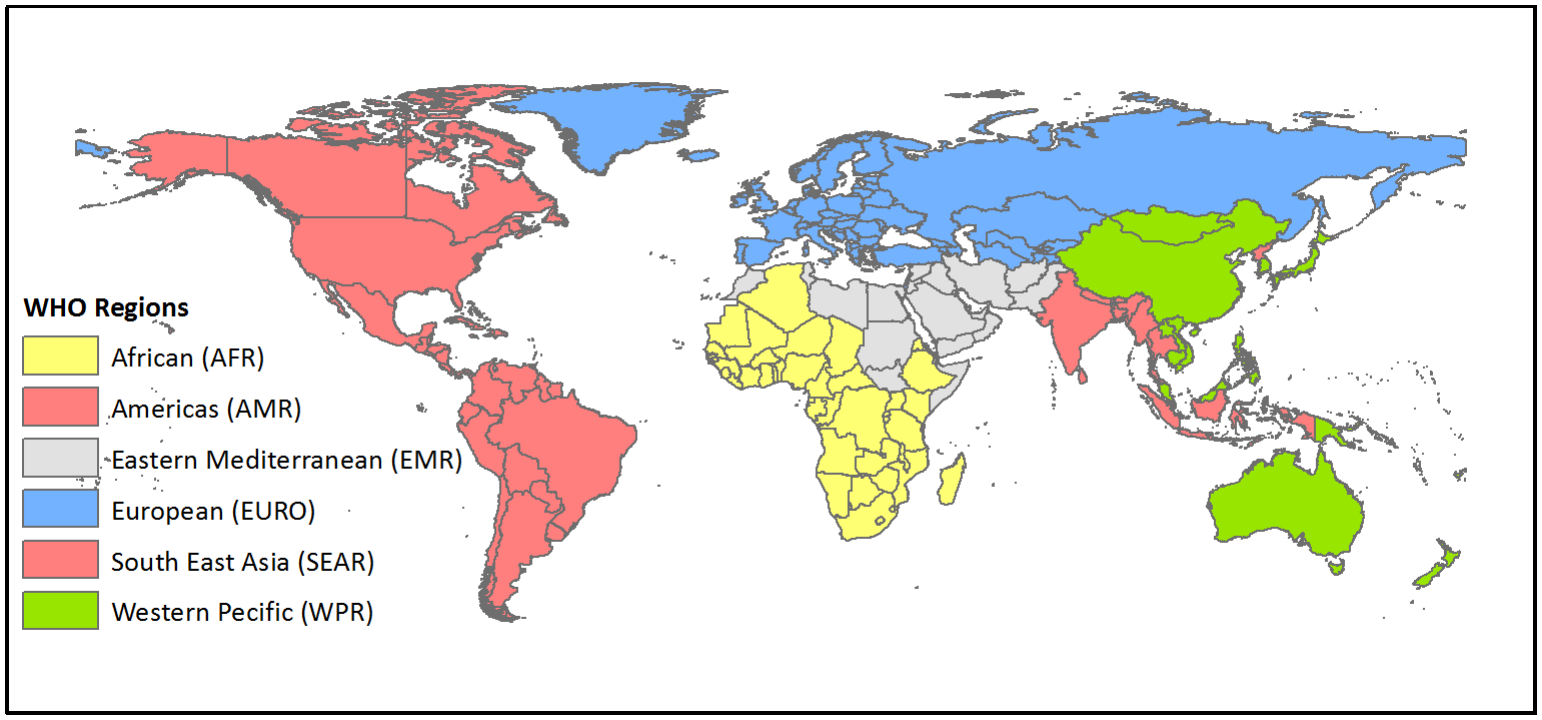
World Health Organization regions.

Countries were stratified based on the six WHO regions (see Fig 1 and Table 1) and five WHO mortality strata: (A) very low child and low adult mortality; (B) low child and low adult mortality; (C) low child and high adult mortality; (D) high child and high adult mortality; and, (E) high child and very high adult mortality [14]. All countries in the A stratum with greater than 95% access to improved sanitation facilities were excluded from the model as the probability of cholera endemicity was assumed to be zero, and ability for secondary transmission of imported cases was also assumed to be zero.

**Table 1:** Incidence rate and case fatality rate for cholera by WHO region.

WHO Region	Incidence rate and source of information		Case fatality rate (%)
	Incidence Rate per 1,000 population at risk	Source of Information	
AFR-D	2.00	Beira, Mozambique [19] and WER data	3.80
AFR-E	4.00	Beira, Mozambique data [19]	3.80
AMR-B: Dominican Republic	5.70	Dominican Republic WER [22–24]	1.40
AMR-B: Jamaica, St. Lucia	0.10	Assumption-based	1.00
AMR-D	25.60	Haiti WER [22–24]	1.20
EMR-D	1.64	Kolkata data [17]	3.20
EUR-B	0.10	Assumption-based	1.00
SEAR-B	0.45	Jakarta data [18]	1.00
SEAR-D	1.64	Kolkata data [17]	3.00
WPR-B	0.10	Assumption-based	1.00

### Endemic and Non-endemic Countries

A spatial regression model was developed to predict whether or not a country had cholera cases for each year from 2008–2012. The model’s outcome variable was the log-transformed annual incidence rate. The incidence rate was calculated based on the number of cases reported in a particular calendar year per UNDP estimated total population in the same calendar year, multiplied by a constant to account for 10% reporting efficiency [2]. For countries with no reported cases, the incidence rate of 0 cases/100,000 population was replaced with 0.001 cases/100,000 population, which is below the threshold for sustained transmission in a population and one log below the lowest observed incidence rates, so that the figures could be log-transformed and included in the model. All United Nations member states, with the exception of countries which were in the “A” mortality stratum and had greater than or equal to 95% of their population with sustainable access to improved sanitation, were included in the model. The predictor variables included in the model were 1) the percent of the population without sustainable access to improved sanitation, and 2) the percent of the population without sustainable access to improved drinking water sources, as defined by UNICEF [12]. Both predictor variables were significantly associated with the outcome variable in a multivariate regression model for every year analyzed from 2008–2012 (p<0.05). The data for the model can be found in supporting information (see S1 Dataset).

The spatial regression was run for each year in GeoDa, which is an open source software for spatial data analysis. Spatial weights were created with first order Queen contiguity (contiguity based on shared border or vertices). Queen contiguity was selected over Rook contiguity (contiguity based on shared border only) because the absolute length of two countries’ shared border was less important than the proximity of the countries.

Initially, we ran ordinary least square (OLS) regression, and based on the results of the regression diagnostics for spatial dependence we chose spatial regression model. A threshold incidence rate of 0.01 cases/100,000 population was established based on the closest match to what we expected the results to be in terms of countries’ classification of endemic, non-endemic, or cholera-free. If the model’s predicted incidence rate exceeded the threshold for a given year in a particular country, that country was determined to have cholera cases in that year.

Based on the results of the model, countries were determined to be endemic, non-endemic, or cholera-free. Countries were defined as cholera endemic if they had predicted cholera cases in at least three of the five years under study (2008–2012). This definition is in line with the WHO Strategic Advisory Group of Experts on Vaccines and Immunization (SAGE) definition of a cholera-endemic country [15]. Non-endemic countries were those with predicted cholera cases in one or two years during the five year study period (2008–2012).

### Population at Risk

The population at risk was determined using the percentage of the population without access to an improved sanitation facility. Though access to improved sanitation facilities is not the only determinant of cholera risk, this indicator was selected as a proxy measure due to the lack of availability of other reliable data at the country level [6]. For India, China, and Indonesia, sub-national population data were collected from each country’s national statistics division [16–18]. For these three countries, the population at risk was calculated based on the percentage of the population without access to improved sanitation in the states (India) or provinces (China and Indonesia) which had reported cholera cases in 2008–2012. Applying an incidence rate to the entire population at risk in these countries would artificially inflate the global burden of cholera; thus, the population at risk was narrowed to the states and provinces which had reported cases of cholera in the 5-year period under study. In these three countries, the national-level estimate for the percentage of the population without access to improved sanitation was used as a proxy for the sub-national estimate, as sub-national-level data on the percentage of the population without access to improved sanitation were not available.

### Estimation of Cholera Cases in Endemic Countries

The annual number of cholera cases for the endemic countries (*vide supra*) were estimated from the population at risk multiplied by population-based cholera incidence rate in the country. Population-based cholera incidence rates were obtained from the Diseases of the Most Impoverished (DOMI) cholera surveillance program in Kolkata, India [19], Jakarta, Indonesia [20], and Beira, Mozambique [21]. These incidence rates were used after a literature review performed to find updated population-based incidence rates for cholera found no recent studies. These data were assumed to still be valid because it is unlikely that cholera incidence rates in these areas changed dramatically between 2005 and 2010. The DOMI figures included both inpatient and outpatient cases of laboratory-confirmed cholera, and were assumed to be representative of country-wide incidence rates in countries in the same WHO mortality stratum [6].

The population-based incidence rate from Beira, Mozambique was applied to the AFR-E countries. Based on previous analysis, it was assumed that the incidence rate in AFR-D countries was half the incidence in AFR-E countries [6]. The Kolkata, India incidence rate was applied to EMR-D and SEAR-D countries. The Jakarta, Indonesia incidence rate was used as the incidence rate for Indonesia (SEAR-B). For Haiti and the Dominican Republic, the average observed incidence rate from 2010–2012 was used for each country respectively. The reported incidence rates in these two countries (Haiti and the Dominican Republic) were assumed to be nearly accurate due to the strength of the cholera surveillance system in these countries leading to a high reporting efficiency, and the high reported incidence rates. All other countries were in stratum B. Based on higher proportions of the population with access to improved sanitation and lower reported incidence rates in these two countries, a 0.1 case/1,000 population at risk incidence rate was applied to countries in the B stratum.

### Estimation of Cholera Deaths in Endemic Countries

The annual number of cholera deaths for the endemic countries were estimated from the number of cholera cases multiplied by the cholera case-fatality rate. As others have previously noted [6], cholera mortality is not limited to a certain age group, and is high among all patients. Due to the rapid dehydration of the cholera cases, many deaths occur before these patients are able to reach a health facility. Therefore, it was assumed that facility-based case fatality rates (CFRs) were underestimating the true population CFRs. Instead of using facility-based CFRs, CFRs were calculated using inverse variance-weighted average CFRs by WHO mortality stratum (Table 1). Information on the CFR computation was previously discussed by Ali et al. [6]. Average observed CFRs from 2010–2012 were used for Haiti and the Dominican Republic. Although these CFRs are likely to underestimate the true CFRs, these were used because we assumed that the reporting efficiency is relatively high in these countries as a result of their strong cholera surveillance systems, and because the CFR may be lower than it was in the past three years at the height of the epidemic in these two countries.

## Results

### Spatial Dependence

The diagnostics for spatial dependence from the OLS model showed considerable spatial dependence (Moran I = 0.3241, p<.0001), with significant Robust Lagrange Multiplier error (p<.0001). However, Robust Lagrange Multiplier Lag did not yield to a value (p = .07), which suggested a spatial error regression model is best fit for the data [22]. Therefore, we applied a spatial error model to predict the incidence for each year. Based on a univariate Moran’s I test with 1^st^ order of neighbor spatial weights, the residuals of the predicted log-transformed incidence rates were spatially random (p = .22).

### Population at Risk

There are 1.3 billion people at risk for cholera in the 69 countries classified as cholera-endemic. Another 99 million persons are at risk in the three countries the model predicted as non-endemic (i.e., Bolivia, Pakistan, and Sri Lanka). There are twenty-three endemic countries that have over 10 million persons at risk (see Table 2 for country classification and country-specific population at risk). India, Nigeria, China, Ethiopia, and Bangladesh are the countries with the highest number of people at risk for cholera. The population at risk by WHO region and mortality stratum is shown in Table 2.

**Table 2:** Country-specific cholera cases and deaths.

WHO Region	Country	Population at risk	Incidence rate/ 1,000	Estimated annual number of cases	Case fatality rate (%)	Estimated annual number of deaths
AFR-D	Nigeria	110,198,368	2.00	220,397	3.80	8,375
AFR-D	Ghana	20,866,095	2.00	41,732	3.80	1,586
AFR-D	Madagascar	17,917,602	2.00	35,835	3.80	1,362
AFR-D	Niger	14,463,309	2.00	28,927	3.80	1,099
AFR-D	Burkina Faso	12,898,436	2.00	25,797	3.80	980
AFR-D	Mali	10,909,050	2.00	21,818	3.80	829
AFR-D	Cameroon	10,518,415	2.00	21,037	3.80	799
AFR-D	Chad	10,197,079	2.00	20,394	3.80	775
AFR-D	Guinea	8,918,347	2.00	17,837	3.80	678
AFR-D	Benin	8,273,524	2.00	16,547	3.80	629
AFR-D	Angola	8,210,632	2.00	16,421	3.80	624
AFR-D	Senegal	6,216,271	2.00	12,433	3.80	472
AFR-D	Togo	5,486,232	2.00	10,972	3.80	417
AFR-D	Sierra Leone	5,004,219	2.00	10,008	3.80	380
AFR-D	Liberia	3,245,552	2.00	6,491	3.80	247
AFR-D	Mauritania	2,670,971	2.00	5,342	3.80	203
AFR-D	Guinea-Bissau	1,269,299	2.00	2,539	3.80	96
AFR-D	Gabon	1,042,669	2.00	2,085	3.80	79
AFR-D	Gambia	537,805	2.00	1,076	3.80	41
AFR-D	Comoros	437,172	2.00	874	3.80	33
AFR-D	Cape Verde	190,164	2.00	380	3.80	14
AFR-D	Sao Tome and Principe	131,889	2.00	264	3.80	10
AFR-E	Ethiopia	68,805,272	4.00	275,221	3.80	10,458
AFR-E	Democratic Republic of the Congo	47,265,282	4.00	189,061	3.80	7,184
AFR-E	United Republic of Tanzania	40,475,997	4.00	161,904	3.80	6,152
AFR-E	Kenya	27,818,252	4.00	111,273	3.80	4,228
AFR-E	Uganda	22,431,561	4.00	89,726	3.80	3,410
AFR-E	Mozambique	19,653,157	4.00	78,613	3.80	2,987
AFR-E	Côte d'Ivoire	14,422,207	4.00	57,689	3.80	2,192
AFR-E	Zimbabwe	7,846,187	4.00	31,385	3.80	1,193
AFR-E	Malawi	7,356,710	4.00	29,427	3.80	1,118
AFR-E	South Sudan	7,356,287	4.00	29,425	3.80	1,118
AFR-E	Zambia	6,872,832	4.00	27,491	3.80	1,045
AFR-E	Eritrea	5,454,101	4.00	21,816	3.80	829
AFR-E	Burundi	4,985,687	4.00	19,943	3.80	758
AFR-E	Rwanda	4,876,529	4.00	19,506	3.80	741
AFR-E	Congo	3,371,606	4.00	13,486	3.80	512
AFR-E	Central African Republic	2,870,948	4.00	11,484	3.80	436
AFR-E	Lesotho	1,486,602	4.00	5,946	3.80	226
AFR-E	Namibia	1,481,698	4.00	5,927	3.80	225
AFR-E	Swaziland	513,054	4.00	2,052	3.80	78
AMR-B	Dominican Republic	1,702,855	5.70	9,639	1.40	138
AMR-B	Jamaica	548,297	0.10	55	1.00	1
AMR-B	Saint Lucia	62,089	0.10	6	1.00	0
AMR-D	Haiti	8,214,012	26.00	210,589	1.20	2,584
EMR-D	Sudan	26,382,481	1.64	43,267	3.20	1,385
EMR-D	Afghanistan	17,890,622	1.64	29,341	3.20	939
EMR-D	Yemen	10,698,614	1.64	17,546	3.20	561
EMR-D	Somalia	7,419,853	1.64	12,169	3.20	389
EMR-D	Djibouti	417,018	1.64	684	3.20	22
EUR-B	Tajikistan	457,640	0.10	46	1.00	0
SEAR-B	Indonesia	5,107,432	0.45	2,298	1.00	23
SEAR-D	India	411,700,175	1.64	675,188	3.00	20,256
SEAR-D	Bangladesh	66,495,209	1.64	109,052	3.00	3,272
SEAR-D	Nepal	18,523,751	1.64	30,379	3.00	911
SEAR-D	Timor-Leste	572,109	1.64	938	3.00	28
SEAR-D	Bhutan	401,486	1.64	658	3.00	20
WPR-B	China	90,838,800	0.10	9,084	1.00	91
WPR-B	Philippines	24,295,524	0.10	2,430	1.00	24
WPR-B	Cambodia	9,911,802	0.10	991	1.00	10
WPR-B	Papua New Guinea	3,772,420	0.10	377	1.00	4
WPR-B	Lao People's Democratic Republic	3,325,771	0.10	333	1.00	3
WPR-B	Solomon Islands	357,984	0.10	36	1.00	0
WPR-B	Vanuatu	101,609	0.10	10	1.00	0
WPR-B	Micronesia (Fed. States of)	77,714	0.10	8	1.00	0
WPR-B	Kiribati	65,488	0.10	7	1.00	0
WPR-B	Marshall Islands	13,107	0.10	1	1.00	0
WPR-B	Palau	6,755	0.10	1	1.00	0
WPR-B	Nauru	3,509	0.10	1	1.00	0
**Total**		**1,264,311,192**		**2,855,714**		**95,284**

### Cholera Cases

**Fig 2:**
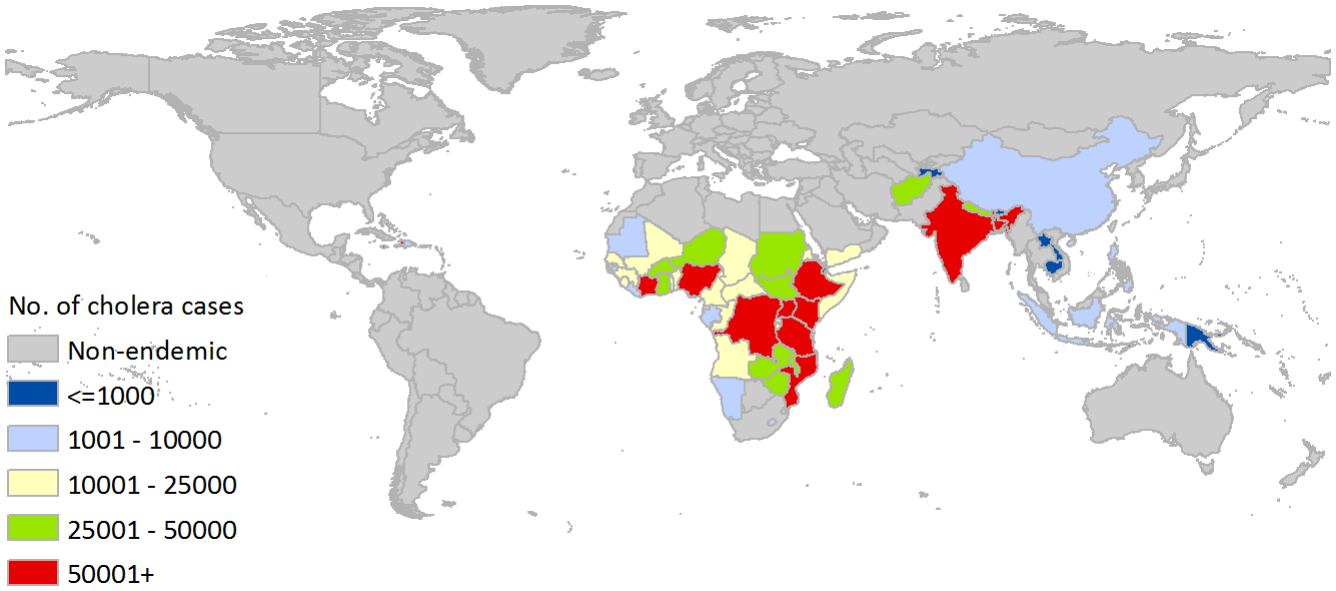
Annual number of cholera cases in endemic countries.

There are an estimated 2.86 million cases of cholera annually in endemic countries. Spatial distribution of the burden of cholera in endemic countries are shown in Fig 2. Countries with estimates of more than 100,000 cases annually include: India, Ethiopia, Nigeria, Haiti, the Democratic Republic of the Congo, Tanzania, Kenya, and Bangladesh (for country-level estimates, see Table 2). The WHO regions with the highest burden of cases are AFR-E, SEAR-D, which includes India and Bangladesh, and AFR-D (Table 3). Haiti alone (AMR-D) has a greater burden of cholera cases than all endemic countries in the B-level mortality stratum combined. The average incidence rate in endemic countries is 2.30 cases/1,000 population at risk per year. Although classified as non-endemic, Pakistan, Bolivia and Sri Lanka were estimated to have a cumulative average of 2,737 cases reported annually.

**Table 3:** Population at risk (in descending order of risk) and estimated number of cases and deaths by WHO region-mortality stratum.

WHO Region-Mortality Stratum	Population at risk	Estimated annual number of cases	Estimated annual number of deaths
AFR-D	259,603,100	519,206	19,730
AFR-E	295,343,969	1,181,376	44,892
AMR-B	2,312,241	9,700	139
AMR-D	8,214,012	210,589	2,584
EMR-D	62,808,588	103,006	3,296
EUR-B	457,640	46	0
SEAR-B	5,107,432	2,298	23
SEAR-D	479,692,729	816,216	24,486
WPR-B	132,770,482	13,277	133
**TOTAL**	**1,246,310,193**	**2,855,714**	**95,283**

### Cholera Deaths

Cholera resulted in approximately 95,000 deaths annually in endemic countries (Table 2). This translates to approximately 7.50 deaths/100,000 population at risk per year in endemic countries. Of the countries with more than 1,000 deaths due to cholera annually, all are in the African Region except for India (SEAR), Bangladesh (SEAR), Haiti (AMR), and Sudan (EMR). Countries in the AFR-E stratum have a disproportionate burden of cholera deaths (see Table 2 for country-specific estimated deaths due to cholera).

### Sensitivity Analyses

Sensitivity analyses were conducted to produce conservative and liberal estimates in order to avoid over- or under-estimating the annual number of cases and deaths in endemic countries as defined above. The sensitivity analyses included modifications to: a) population at risk; b) incidence rates; and, c) case fatality rates. The first sensitivity analysis established a conservative estimate for population at risk using the fraction of the population with sustainable access to improved water instead of the fraction of the population with sustainable access to improved sanitation. When population at risk was calculated using access to improved water, the total number of cholera cases was 1.20 million annually, and the total number of deaths due to cholera was 44,000 annually. The liberal estimate for population at risk used the entire population of Indonesia, India, and China (i.e., all states/provinces) to calculate the at risk population in these countries. This liberal estimate increased the total estimated number of annual cholera cases and deaths to 3.04 million and 104,000, respectively (Table 4).

**Table 4:** Sensitivity analyses results.

Parameter Used	Total annual Cases	Total Annual Deaths
Population at risk (Conservative)	1,203,545	43,661
Population at risk (Liberal)	3,038,420	103,637
Estimated IR * 50% (Conservative)	1,335,874	46,507
Estimated IR * 150% (Liberal)	4,007,622	139,521
1% CFR (Conservative)	2,855,714	21,379
5% CFR (Liberal)	2,855,714	142,786

The second sensitivity analysis applied incidence rates of 50% and 150% of the original incidence rate. When incidence rates were halved (conservative estimate), the total annual numbers of cholera cases and deaths were 1.36 million and 47,000, respectively. The liberal estimate of the incidence rate predicted 4.01 million cases and 140,000 deaths annually. The third sensitivity analysis assumed a 1% CFR (conservative estimate) or a 5% CFR (liberal estimate) in all endemic countries. In this analysis, the total number of cholera cases remained the same, but the estimated annual number of deaths due to cholera ranged from 21,000 under the conservative CFR assumption to, 143,000 under the liberal CFR assumption (see S2 Dataset for country-specific estimates of the sensitivity analyses in supporting information).

## Discussion

We estimate that there were 2.9 million cases (uncertainty range: 1.3 to 4.0 million) of cholera annually in 69 cholera-endemic countries and 95,000 deaths (uncertainty range: 21,000–143,000) between 2008 and 2012. The total estimates are similar to the global burden estimates from 2000–2008 [6], however the distribution is different. Our study showed that Sub-Saharan Africa accounted for 60% and South-East Asia accounted for 29% of the global burden of cholera cases between 2008 and 2012. Our findings highlight the fact that cholera remains an important public health issue in more than one-third of the countries of world.

Precise estimates of country-level cholera burden remain a challenge for a number of reasons. First is the lack of standard reporting of cholera cases and deaths. The average number of cases and deaths reported by the WHO and other sources from 2008–2012 was 331,337 and 6,335, respectively, which was only 11.6% of the estimated number of cases and 6.6% of the estimated number of deaths. Cases and deaths that do not present to health facilities are not included in the reports and this underestimates the true burden of cholera. In a study in Kenya, there were 46% more cholera cases and 200% more deaths identified through active case finding. Due to the rapid onset of dehydration, death may ensue particularly in areas where accessing health care may be limited by distance, lack of transport or cost [23].

The spatial regression model developed for this global burden estimation predicted the presence of cholera in a country based on the observed incidence rate, the proportion of the population with sustainable access to an improved sanitation facility, the proportion of the population with sustainable access to an improved water source, and a spatial weight which takes into consideration the cholera incidence rates of the neighboring countries. This approach allows for burden estimation in countries which may not report any cases of cholera. The total number of countries estimated to be cholera-endemic is 69. This figure is similar to the total number of countries which reported cholera cases in 2008–2012, of which, 42 would be classified as endemic and another 22 would be classified as non-endemic based on reported cholera cases. The estimates produced by this study are slightly higher compared to the previous global burden study (2.9 million vs 2.8 million cases and 95,000 vs 91,000 deaths), and closer to the WHO estimates of 3 to 5 million cases and 100,000 to 120,000 deaths annually [24].

The regression diagnostics suggest that error of the model are spatially correlated. Thus, spatial dependence enters through the errors in our model and not through the systematic component of the model as the case of spatial lag model. Such a model focuses on estimating the parameters for the independent variables of interest in the systematic part of the model, and disregards the possibility that the observed correlation may reflect something meaningful about the data generation process [25]. The residuals of the predicted log-transformed incidence rates from the spatial regression model were spatially random (p = 0.22) suggesting that model adequately addressed spatially correlated error in the model. It also indicates that important independent variables were included in the model and the underlying spatial process that may induce spatial autocorrelation in some of the variables was not missing in the model.

There were several limitations in this global burden estimation. There were a few countries which would be classified as endemic if classification were based solely on reported cases, but our model predicted them to be non-endemic (i.e., Pakistan) or cholera-free (i.e., Malaysia, Myanmar, Iran, Thailand, and Vietnam). This misclassification is due in part to the very low observed incidence rates in these countries, and in part due to the higher proportions of the population with access to improved sanitation and safe water sources than would be expected in cholera-endemic countries. This misclassification may mean that the burden of cholera cases in these countries is regionally disparate or disproportionately affects a small subset of the population. Conversely, some countries for which there is reasonable certainty that they are cholera-free were classified as endemic by our model (i.e., St. Lucia, Jamaica, and Tajikistan). These countries have high proportions of their populations without access to improved water or improved sanitation, and thus highly likely to become endemic should cholera be introduced from outside. Overall, the three countries assumed to be cholera-free which were classified as cholera-endemic added only 107 cases annually to the global burden.

Another limitation of this study is the threshold definition used to determine if a country was predicted to have cholera cases in a given year. The threshold used (0.01 cases/100,000 total population) was selected based on its accuracy in classifying countries known to be cholera-endemic or cholera-free. Due to the spatial heterogeneity of cholera in some of the larger countries, a country-wide threshold may not accurately predict whether the entire country was cholera-free or cholera-endemic in a given year. A further limitation of this study is the inability to elucidate the highest-risk groups within a country due to the lack of accurate age-specific incidence rates and CFRs. Others have recognized that in many countries, children under five are disproportionately at risk for cholera [6]. This should be taken into consideration when decision-makers are planning cholera control programs.

In the absence of reliable country-level incidence rates and CFRs, these rates often had to be taken from other countries within the WHO region or mortality stratum. Spatial and temporal heterogeneity of cholera transmission also means that the country-level burden is rarely constant from year to year, especially in countries with moderate incidence rates. To address this limitation, sensitivity analyses were conducted to generate uncertainty ranges for global estimates of cases and deaths.

Finally, the size and socio-economic diversity in the populations of India, China, and Indonesia make predicting the burden in these countries challenging yet critical to the total global estimate. For these countries, the states or provinces at risk were considered to be only those with reported cases between 2008 and 2012. The incidence and case fatality rates were applied only to these states or provinces. For modeling purposes, the sub-national administrative boundaries were used so that the spatial model would not falsely weigh countries which neighbor cholera-free states or provinces. However, the model is not precise enough to estimate sub-national incidence rates, as the access to water and sanitation data are not available at sub-national levels.

This updated global burden estimation is important for policy and public health decision-makers in countries which may not know of the cholera burden in their countries due to challenges in surveillance and case reporting. The updated findings are equally important for decision-makers who are involved in building the OCV stockpile, as it provides an estimate of the size of the demand for the vaccine. The findings are also important for those who are responsible for requesting OCV for their country, either from the manufacturer or from the WHO’s stockpile, and for members of the International Coordinating Group, who are responsible for managing the use of the OCV stockpile. More accurate data on country-level and global-level cholera burden will allow decision-makers to more effectively allocate resources to the countries with the greatest need.

Our findings show that the cholera burden remains high. Efforts for an integrated approach to cholera control are vital to prevent further spread of the disease and mitigate the resulting mortality and morbidity from this deadly disease.

## Supporting Information

S1 Dataset The data used in the model. Variable names are self-explanatory. Additional information related to the variables are given at the bottom of the table.
(XLSX)

S2 Dataset Data and the results of the sensitivity test by countries. (XLSX)
